# Retinal Microvascular and Orbital Structural Alterations in Thyroid Eye Disease

**DOI:** 10.3390/jcm15010323

**Published:** 2026-01-01

**Authors:** Vera Jelušić, Ivanka Maduna, Dubravka Biuk, Zdravka Krivdić Dupan, Josip Barać, Nikolina Šilješ, Laura Jelušić, Tvrtka Benašić, Jelena Juri Mandić

**Affiliations:** 1 Department of Ophthalmology, University Hospital Center Osijek, 31000 Osijek, Croatia; verajelusic@gmail.com (V.J.); dubravka.biuk@gmail.com (D.B.); jbarac@mefos.hr (J.B.); tvrtka@gmail.com (T.B.); 2 Faculty of Medicine Osijek, Josip Juraj Strossmayer University of Osijek, 31000 Osijek, Croatia; zdravka.krivdic@gmail.com; 3 Health Center of Osijek-Baranja County, 31000 Osijek, Croatia; 4 Department of Radiology, University Hospital Center Osijek, 31000 Osijek, Croatia; nikolina.grbavac91@gmail.com; 5 Department of Cardiology, University Hospital Center Osijek, 31000 Osijek, Croatia; laura.jelusic@kbco.hr; 6 School of Medicine, University of Zagreb, 10000 Zagreb, Croatia; jmiklau@gmail.com; 7 Department of Ophthalmology, University Hospital Center Zagreb, 10000 Zagreb, Croatia

**Keywords:** biomarkers, magnetic resonance imaging, optical coherence tomography angiography, thyroid eye disease

## Abstract

**Background/Objectives:** Thyroid eye disease (TED) can lead to structural and microvascular changes in the orbit and retina. This study aimed to investigate the associations between Clinical Activity Score (CAS), orbital magnetic resonance imaging (MRI) measurements, and retinal microvascular changes in TED patients. **Methods**: This cross-sectional study included 38 patients (76 eyes) with TED. Each patient underwent a comprehensive ophthalmological evaluation, CAS assessment, and a detailed medical history. Optical coherence tomography angiography (OCTA) was performed to quantify vessel density (VD) in the superficial and deep capillary plexus (SCP and DCP). Exophthalmos, extraocular muscle thickness and orbital fat thickness were measured on MRI scans to evaluate structural changes. Laboratory analyses included thyroid hormone levels, thyrotropin receptor antibodies (TRAb), anti-thyroid peroxidase antibodies (anti-TPO), and lipid profile. **Results**: Active TED patients (CAS ≥ 3) had significantly higher TRAb levels (*p* < 0.001), while anti-TPO did not differ between groups. Active eyes showed significantly higher DCP VD in the whole image (*p* = 0.013), parafovea (*p* = 0.012), and perifovea (*p* = 0.009) across all quadrants, with no difference in SCP or the foveal avascular zone (FAZ). In linear mixed model regression analyses, after adjusting for previous glucocorticosteroid therapy, higher triglycerides, greater medial rectus thickness, and whole-image DCP VD independently predicted higher CAS values (R^2^ = 42, *p* < 0.001). After adjusting for age and sex, CAS remained significantly positive predictor of DCP VD in the parafovea (R^2^ = 0.22, *p* < 0.001). **Conclusions**: Changes in DCP VD reflect TED activity and structural orbital involvement.

## 1. Introduction

Thyroid eye disease (TED) represents the orbital manifestation of autoimmune thyroid disorders, most frequently associated with Graves’ hyperthyroidism (GH) [1,2]. It is estimated that 30% of patients with GH develop clinically manifest TED, with a higher incidence in women, but severe forms are more common in males [3,4,5]. It is a heterogeneous condition with diverse clinical presentations that can lead to significant disfigurement [1]. The condition is characterized by varied clinical manifestations involving the orbital and periocular soft tissue, including eyelid erythema and edema, conjunctival hyperemia, proptosis, and diplopia, reflecting both inflammatory and congestive processes. In severe cases, it can lead to loss of vision and blindness caused by compressive optic neuropathy, which occurs in approximately 3% of patients [1,2]. The exact mechanism of TED is still not fully understood, but it is assumed that autoimmune-mediated inflammation, inflammatory cell infiltration, activation of orbital fibroblasts, and hemodynamic changes in the orbit play a significant role [6,7]. Histologically, the disease is manifested by increased lymphocyte and macrophage infiltration, fibroblast proliferation, and accumulation of extracellular matrix, which leads to enlargement of extraocular muscles and orbital fat tissue [8]. Increased intraorbital pressure resulting from the expansion of orbital soft tissues can lead to venous congestion, elevated episcleral venous pressure, and disturbances in choroidal blood flow due to increased vascular resistance [9,10,11]. Identification and monitoring of disease activity are essential to prevent irreversible visual impairment. Clinical activity and severity of TED determine the treatment modality according to the 2021 European Society for Graves’ Orbitopathy (EUGOGO) guidelines. Currently, the assessment of TED activity is carried out qualitatively, while CAS (Clinical Activity Score) remains the most widely applied qualitative tool for assessing TED activity and progression [2,12]. Magnetic resonance imaging (MRI) is routinely used in clinical practice as an adjunctive tool to assess disease activity and provide detailed anatomical information, including extraocular muscle thickness, degree of exophthalmos, and potential optic nerve compression [13]. As an advanced, non-invasive technique, optical coherence tomography angiography (OCTA) allows for high-quality imaging of the retina, revealing detailed structural features and microvasculature [14]. Although several imaging modalities and measurement techniques are available, a clear lack of standardization persists across studies and clinical practice. This study aimed to evaluate the relationship between orbital structural changes and retinal microvascular alterations in patients with TED. Additionally, we focused on identifying clinical, biochemical, and imaging predictors of disease activity, with a particular focus on retinal vessel density (VD), extraocular muscle thickness and orbital fat thickness, as potential quantitative, objective markers of inflammation and disease activity.

## 2. Materials and Methods

This cross-sectional study was conducted at the Department of Ophthalmology, University Hospital Center Osijek, Croatia, from December 2024 to June 2025. The study was conducted in accordance with the principles of the Declaration of Helsinki and approved by the Ethics Committee of the University Hospital Center Osijek. Written informed consent was obtained from all patients before their inclusion. The study included adult Caucasian patients (≥18 years) of both sexes with a confirmed diagnosis of TED treated at our department. Exclusion criteria were anterior segment pathology (e.g., central keratopathy), primary open- or closed-angle glaucoma, posterior segment disorders (e.g., diabetic retinopathy, age-related macular degeneration, retinal vascular occlusion), previous ocular trauma or uveitis, refractive errors exceeding ±6.0 Diopter sphere or ±3.0 Diopter cylinder, media opacities that compromised angio optical OCTA image quality, intravenous glucocorticosteroids therapy 3 months prior the inclusion, and any contraindication for orbital MRI.

### 2.1. Clinical Evaluation

All included participants underwent a detailed medical history review and a comprehensive ophthalmologic examination, including best-corrected visual acuity (BCVA), slit-lamp biomicroscopy, pupillary reactions, intraocular pressure (IOP) measurement using Goldman applanation tonometry in primary and upward gaze, exophthalmos measurement using Hertel exophthalmometer, and dilated fundus examination. All Hertel measurements were performed by the same examiner. Disease activity in TED patients was assessed according to the EUGOGO guidelines using CAS [4]. Based on CAS, eyes were classified as active (CAS ≥ 3) or inactive (CAS < 3). The following data were collected: age, sex, duration of GH and TED, smoking status, established diagnosis of arterial hypertension, history of radioiodine (RAI) therapy and antithyroid drug use and serum concentrations of thyroid-stimulating hormone (TSH), free triiodothyronine (fT3), free thyroxine (fT4), thyroid-stimulating hormone receptor antibodies (TRAb), and thyroid peroxidase antibodies (anti-TPO), total cholesterol, low-density lipoprotein (LDL), high-density lipoprotein (HDL), and total triglycerides. Lipid profile data were available for 29 participants.

### 2.2. OCTA Imaging

OCTA was performed using the OptoVue XR Avanti device (OptoVue Inc., Fremont, CA, USA) equipped with the split-spectrum amplitude-decorrelation angiography (SSADA) algorithm (version 2014.2.0.90). The 6 × 6 mm^2^ Angio Retina protocol was used for all scans. Retinal microvasculature was analyzed in the superficial and deep capillary plexuses (SCP and DCP), automatically segmented by the software. The SCP was defined as 3–15 μm below the internal limiting membrane, and the DCP as 15–70 μm below it. VD was measured for each plexus in nine standardized Early Treatment Diabetic Retinopathy Study (ETDRS) regions: central fovea, parafovea, and perifovea (each subdivided into superior, inferior, nasal, and temporal quadrants). The foveal avascular zone (FAZ) area was also recorded.

### 2.3. Orbital MRI Imaging

MRI images acquired using 1.5T and 3T scanners (Siemens Magnetom Avanto Fit and Magnetom Skyra, Siemens Healthineers, Erlangen, Germany) during TED evaluation were reviewed manually by an experienced radiologist using the PACS imaging software (Sectra IDS7, Version 24.1, Linköping, Sweden). The radiologist was blinded to patient outcomes, and measurements were performed on T1- and T2-weighted images in both coronal and axial planes. The maximal thickness of each of the four rectus muscles (superior, inferior, medial, and lateral) was measured in millimeters. Medial and lateral rectus thicknesses were obtained in axial sections, whereas superior and inferior rectus muscles were measured on coronal images. For each muscle, measurements were made at its widest point in the midsection. Orbital fat thickness was determined as the maximal distance between the medial wall of the orbit and the lateral margin of the medial rectus muscle. Exophthalmos was quantified as the perpendicular distance between the corneal apex and the interzygomatic line.

### 2.4. Statistical Analysis

Categorical data were presented as absolute and relative frequencies. Continuous variables were expressed as mean (standard deviation [SD]) or median (interquartile range [IQR]), depending on distribution. Differences between categorical variables were analyzed using the Chi-square or Fisher’s exact test, as appropriate. Continuous variables were assessed for normality using the Shapiro–Wilk test and histogram analysis. For non-normal distributions, group comparisons were performed using the Mann–Whitney U test, and results are presented with Hodges–Lehmann median difference estimates and their 95% confidence intervals (CI). For normally distributed data, group comparison was analyzed using Student’s *t*-test with Hedges’ g mean difference and its 95% CI. For patient-level parameters, patients were classified as active if at least one eye had CAS ≥ 3 and inactive if both eyes had CAS < 3. Spearman’s correlation analysis was performed using data from the right patient’s eye to assess associations between clinical, biochemical, microvascular, and MRI-derived parameters. Additionally, a generalized mixed effects model was used to analyze and compare differences in eye-level parameters. The model included a random intercept for patient and fixed effects for the group (active vs. inactive TED group). Univariate and multivariate mixed-effect regression analyses were conducted to identify predictors of retinal microvascular alterations and disease activity in patients with TED. Variables showing significant univariate associations were entered into a hierarchical linear mixed-effects model, with CAS and parafoveal DCP VD as dependent variables. Multicollinearity was assessed using the variance inflation factor (VIF), and no issues were detected. Diagnostic performance of extraocular muscle thickness, DCP VD parafoveal, and the combined prediction model was assessed using receiver operating characteristic (ROC) curve analysis, with area under the curve (AUC), optimal cut-off values (Youden’s Index), and corresponding sensitivity and specificity calculated. Predicted values used in the ROC analysis were obtained from the linear regression model. All *p*-values were two-tailed, with statistical significance set at *p* = 0.05. Data analysis was performed using JASP software (version 0.19.3; JASP Team, 2024) and The jamovi project (2025). jamovi (Version 2.6) [Computer Software].

For sample size planning in this study, a medium-to-large effect size (Cohen’s d = 0.64) was conservatively selected, with a significance level of 0.05 and a statistical power of 0.80. Based on these assumptions, the estimated minimum required sample size for testing the differences between two groups was 80 participants (40 per group). The study included 76 participants, which provides nearly the same statistical power (≈0.79). Due to the relatively small sample size, we additionally reported Hedges’ g as a small-sample–adjusted effect size, as well as the Hodges–Lehmann estimate, together with their corresponding 95% CI. These supplementary measures provide more robust and distribution-independent estimates of group differences under conditions of limited sample size. For hierarchical regression analysis with f^2^ = 0.15 required sample size was 68 participants, calculated using G*Power software 3.1.9.7 (Heinrich-Heine-Universität Düsseldorf, Düsseldorf, Germany).

## 3. Results

A total of 76 eyes from 38 patients with TED were included in the analysis. The mean age was 50.08 (11.59) years (range 25–78), and 71.05% of patients were female. The mean treatment duration with antithyroid medication (athyriazol) was 13.24 months (SD 10.22, range 0–34). The mean CAS was 1.57 (SD 1.34, range 0–5). Sixteen eyes (21.05%) were classified as active (CAS ≥ 3). The two groups, active and inactive, were comparable regarding age and sex distribution. There was no significant association between thyroid functional status (euthyroid, hyperthyroid, hypothyroid) and CAS category (*p* = 0.466). No significant differences were found in VD of the SCP across any analyzed region—whole image, fovea, parafovea, or perifovea—between inactive and active TED group (*p* = 0.683, 0.668, 0.914, and 0.615, respectively), nor in the FAZ area (*p* = 0.789). Significant differences in DCP VD were observed in the parafoveal (*p* = 0.012) and perifoveal regions (*p* = 0.009). When analyzed by quadrant, active eyes showed higher VD in all parafoveal and perifoveal subregions (nasal, temporal, superior, and inferior; all *p* < 0.05). In generalized mixed-effects models accounting for inter-eye clustering, higher DCP VD was significantly associated with active TED. The whole-image DCP VD was a strong predictor of activity (β = 0.62 ± 0.26, OR 1.85, 95% CI 1.12–3.06, *p* = 0.016). Parafoveal DCP VD showed a comparable association (β = 0.58 ± 0.23, OR 1.78, 95% CI 1.13–2.81, *p* = 0.012). Perifoveal DCP VD was also significant but with a smaller effect size (β = 0.42 ± 0.19, OR 1.51, 95% CI 1.04–2.20, *p* = 0.030). Foveal DCP VD was not associated with TED activity (*p* = 0.192). All models demonstrated a very high intraclass correlation (ICC 0.95–0.98). In the generalized mixed model, SCP VD showed no difference between groups (*p* = 0.781, 0.561, 0.845, and 0.935). Superior rectus thickness was the only MRI parameter that showed a statistically significant association with TED activity in the mixed-effects model (β = 0.72 ± 0.35, OR 2.05, 95% CI 1.03–4.11, *p* = 0.042). The ICC for SR was 0.36. Baseline demographic and laboratory characteristics are presented in Table 1. Clinical, angiographic, and MRI characteristics are presented in Table 2.

Spearman’s correlation analysis revealed several notable associations among the examined variables (Table S1). Strong positive correlations were observed between the OCTA VD parameters (*p* < 0.001), indicating substantial shared variance among these measures. Functional and structural parameters demonstrated more modest relationships. Fat tissue thickness correlated positively with medial rectus thickness (ρ = 0.40, *p* = 0.013) and inferior rectus thickness (ρ = 0.36, *p* < 0.027). Medial rectus thickness correlated positively with lateral rectus thickness (ρ = 0.56, *p* < 0.001), while inferior rectus thickness showed significant associations with MR (ρ = 0.60, *p* < 0.001). Several clinical features also showed significant relationships: exophthalmos correlated positively with medial rectus thickness (ρ = 0.38, *p* = 0.019), lateral rectus thickness (ρ = 0.39, *p* = 0.014), superior rectus thickness (ρ = 0.37, *p* = 0.021), and inferior rectus thickness (ρ = 0.39, *p* = 0.015). Among biochemical variables, triglycerides correlated with cholesterol (ρ = 0.47, *p* = 0.014). Age demonstrated significant negative correlations with DCP VD whole image (ρ = −0.54, *p* < 0.001) and DCP VD perifovea (ρ = −0.46, *p* = 0.004). GH duration correlated positively with fat tissue thickness (ρ = 0.37, *p* = 0.023). CAS was weakly associated with cholesterol (ρ = 0.37, *p* = 0.050), but showed no significant correlations with OCTA vascular parameters.

In univariate linear mixed model analyses with parafoveal DCP VD as the dependent variable, no significant associations were found with sex, duration of thyroid-associated orbitopathy, duration of hyperthyroidism, TRAb titers, serum triglycerides, or MRI-derived orbital parameters. Age was associated with lower parafoveal DCP VD values (B = −0.11, *p =* 0.042).

In hierarchical linear mixed model with parafoveal DCP VD as the dependent variable, the first model, including age and sex, explained 12% of the variance, with age emerging as a significant negative predictor. In Model 2 CAS significantly improved model fit, showing a strong positive association with DCP VD, while controlling for age and sex. In the final model (Model 3), which included exophthalmos, CAS remained a significant positive predictor, while exophthalmos demonstrated a negative association with DCP VD. The final model accounted for 26% of the variance in parafoveal DCP VD (Table 3). No multicollinearity issues were detected.

In univariate linear mixed model analyses, higher TRAb titers (β = 0.02, *p* = 0.046), total cholesterol (β = 0.28, *p* = 0.037), triglycerides (β = 0.43, *p* = 0.021), greater DCP VD in the whole image (β = 0.06, *p* = 0.025), and increased medial (β = 0.39, *p* = 0.002) and inferior rectus muscle thickness (β = 0.35, *p* < 0.001) were significantly associated with higher CAS. Age, sex, and orbital fat tissue thickness were not significant predictors (all *p* > 0.05). A hierarchical linear mixed-effect regression with CAS as the outcome is presented in Table 4. Due to strong collinearity between medial and inferior rectus muscle thicknesses, only medial rectus thickness was included in the final regression models. Model 2 (biochemical block: TRAb, triglycerides) while controlling for previous intravenous glucocorticosteroid therapy, explained 31% of the variance. The final Model 3, which additionally included DCP VD in the whole image and medial rectus thickness, explained 42% of the variance. In the final model, triglycerides, medial rectus muscle thickness, and DCP VD in the whole image were independent positive predictors of CAS, whereas TRAb was no longer significant (β = 0.01, *p* = 0.557). No multicollinearity issues were detected.

**Figure 1:**
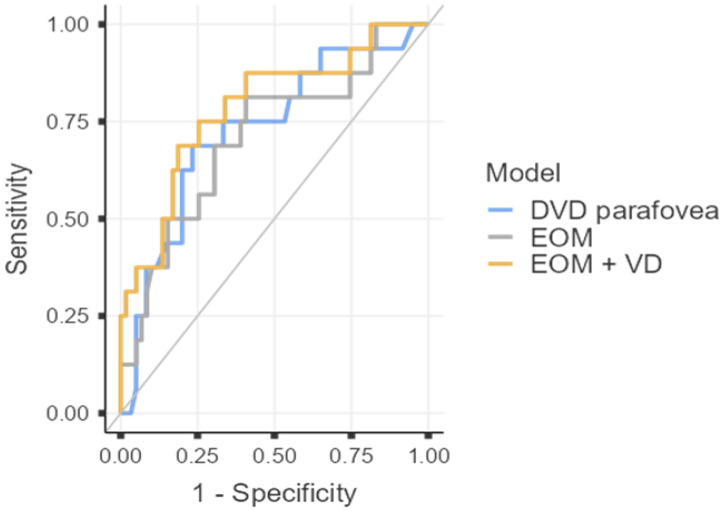
Diagnostic Performance of Structural and OCTA Parameters for Identifying Active TED. ROC curve analysis for extraocular muscle (EOM) thickness sum, parafoveal deep capillary plexus vessel density (DVD parafovea), and the combined predictive model (EOM + VD). The combined model yielded the highest AUC (0.79), with specificity of 81.4% and overall diagnostic accuracy of 78.7%.

ROC analysis demonstrated that both structural (extraocular muscle thickness) and microvascular parameters (DCP VD in the parafovea) were individually associated with disease activity in TED. Sum of extraocular rectus muscle thickness showed moderate diagnostic ability (AUC = 0.71, 95% CI 0.56–0.86, *p* = 0.007), with high sensitivity (81.3%) but limited specificity (59.3%). DCP parafoveal VD performed slightly better (AUC = 0.73, 95% CI 0.58–0.87, *p* = 0.002), achieving balanced sensitivity (68.8%) and specificity (76.7%). The combined model incorporating both parameters achieved the highest discriminatory performance (AUC = 0.79, 95% CI 0.65–0.92, *p* < 0.001), with 81.4% specificity and an overall diagnostic accuracy of 78.7%. The positive likelihood ratio of 3.69 indicates meaningful rule-in capability for active TED, while a negative likelihood ratio of 0.38 reflects a good ability to exclude disease activity (Figure 1).

## 4. Discussion

TED is an autoimmune disorder most frequently associated with GH. It is characterized by remodeling of orbital soft tissues, including extraocular muscle enlargement and orbital fat expansion, which can compress intraorbital structures and alter ocular blood flow [8]. The present study investigated the relationship between orbital structural alterations and retinal microvascular changes in patients with TED. A total of 76 eyes from 38 TED patients were analyzed. Consistent with previous epidemiological reports, we observed a close temporal relationship between the onset of GH and the development of ocular manifestations of TED [15].

The median CAS was 2, which was consistent with earlier studies in TED patients that have reported a much higher prevalence of mild and inactive forms, probably due to earlier referral to specialized TED centers [5]. It should also be noted that we enrolled patients with early-onset TED and patients with TED of longer duration. Advancing age, cigarette smoking, and previous RAI treatment have been identified as significant risk factors influencing both the development and clinical severity of TED [16]. In our cohort, only 4 patients (10.53%) had a history of RAI therapy, which may have contributed to the overall lower CAS scores observed. Also, we observed no difference in smoking status between the active and inactive groups. Similar results have been previously reported [17]. The absence of observed differences may partly be influenced by unmeasured passive smoking, a recognized risk factor, and insufficient data on smoking intensity and duration [18,19].

Emerging evidence indicates that TED affects ocular blood flow. However, recent OCTA studies assessing retinal microvascular alterations in TED have reported heterogeneous results and the precise link between retinal microvascular alterations and disease progression is not fully understood. Several authors demonstrated increased macular VD in the active stage, which may reflect inflammatory hyperperfusion, microvascular dilatation and vascular congestion [20,21], whereas others described capillary rarefaction, particularly within the parafoveal region during active disease [22,23,24]. Reduced macular perfusion may result from orbital congestion and increased endothelin-1 released by retinal endothelial cells, leading to microvascular constriction [25,26]. Several possible factors may lead to increased retinal VD in patients with TAO, including hyperthyroidism, increased cardiac output and orbital inflammation secondary to immune response [27,28,29,30]. Hyperthyroidism is known to reduce systemic vascular resistance and diastolic blood pressure, while increasing cardiac output, systolic pressure, and heart rate [31], which together may increase ocular perfusion. In our study, we did not identify any significant differences in thyroid functional status or hypertension prevalence between active and inactive TED groups. Perri et al. observed significantly higher retinal blood flow in TED patients compared with controls. Also, they found a positive association between extraocular muscle index and retinal perfusion [30]. Ye et al. observed increased SCP VD and DCP VD in TED compared with healthy controls [20]. In the present study, we found a significant increase in DCP VD across the whole image and in all parafoveal and perifoveal subregions of active eyes, while no differences were observed in SCP VD between groups. Similarly, Dogan et al. demonstrated increased DCP VD in active TED, with no difference between hyperthyroid and euthyroid patients, suggesting that orbital inflammation was higher in active patients, causing an increase in DCP VP [32]. Alp et al. argued that elevated ocular blood flow velocity is secondary to orbital inflammation [33]. Differences between retinal microvascular layers may reflect anatomical and physiological distinctions, as retinal circulation primarily supplies the retinal SCP, whereas the DCP also depends on choroidal perfusion [14,30]. Liang et al. further compared hyperthyroid patients with active TED and without TED, finding higher VD in active TED, supporting microvascular dilatation due to orbital inflammation changes [21]. Some evidence also indicates higher parafoveal VD in inactive TED compared with controls, possibly due to compensatory vascular post-inflammatory remodeling [34]. In contrast, Mihailović et al. reported a reduction in parafoveal SCP VD among inactive patients compared to healthy controls, with no difference observed in the DCP [24], further underscoring the variability between studies. Jamshidian Tehrani et al. and Abrishami et al. both reported lower DCP VD in active patients. Jamshidian Tehrani et al. also observed a reduction in SCP VD, while Abrishami et al. found no difference [23,35]. Yu et al. noted decreased SCP VD in the temporal and inferior parafoveal regions of active eyes but did not assess the DCP [22]. Xu et al. also found that while SCP perfusion remained comparable between active and inactive disease, DCP perfusion was significantly reduced in active cases; compared with healthy controls, both active and inactive groups showed regional reductions, most pronounced in the active phase [14]. Recent studies reported enlarged FAZ area in both active and inactive disease compared with healthy eyes [14,22]. In our study, we did not find a difference in FAZ area between active and inactive eyes, which aligns with other studies [21,24,36]. Overall, these findings indicate that retinal microvascular changes in TED are dynamic and phase-dependent. The observed discrepancies may be attributed to variations in demographic factors, axial length, disease activity and scoring methods, or OCTA imaging protocols and software algorithms [37]. It is also important to consider potential ethnic variability, as differences in orbital anatomy and disease expression across populations may partly account for inconsistencies between studies [38]. In the correlation analysis, parafoveal DCP VD was significantly associated with age, whereas no association was observed with CAS, implying the age-related microvascular decline [39]. However, in the mixed regression models, CAS emerged as a significant positive predictor of DCP VD even after adjustment for age and sex, indicating that inflammatory activity exerts an independent effect on retinal perfusion. These findings suggest that DCP VD reflects TED activity independently of age. Furthermore, after adjusting for age, sex and CAS, exophthalmos remained an independent negative predictor of parafoveal DCP VD. The possible explanation is that orbital expansion contributes to reduced retinal perfusion, likely through compression of the posterior pole rather than inflammatory activity. This is supported by the observation that orbital decompression improves macular blood perfusion [40]. Both correlation and regression analyses demonstrated substantial collinearity among extraocular muscle thickness parameters, showing simultaneous involvement of multiple muscles during both acute inflammation and later fibrotic remodeling [41]. Fat tissue thickness was not significantly associated with exophthalmos in our cohort, although previous reports have shown expansion of both fat and muscle tissue in relation to proptosis [42,43]. In our study, fat tissue thickness correlated with medial rectus thickness; therefore, the lack of association with proptosis may reflect the relatively small sample and limited variability in proptosis. Since exophthalmos was negatively associated with DCP VD, in accordance with recent studies [17], its confounding effect may partially mask the vascular impact of inflammatory activity contributing to heterogeneity between published results. Taken together, these findings indicate that inflammatory and structural components of TED exert opposing effects on retinal microcirculation and support the value of OCTA-based microvascular metrics in TED evaluation. Given the modest degree of proptosis (Hertel exophthalmometry mean active vs. inactive: 20 [3.05] vs. 17.2 [3.86]) in the present sample, possibly related to relatively low CAS scores, we were unable to fully separate inflammatory effects from the mechanical restriction mechanism and demonstrate its interplay. Furthermore, Alkhadrawi et al. showed that orbital fat volume did not progressively increase with disease severity and patients with mild TED tended to have higher fat volumes, while extraocular muscle volume showed a marked increase from healthy controls to those with mild and severe disease [1]. Similarly, the correlation of fat tissue thickness with GH duration in our study may imply comparable chronic changes. Future studies with larger samples and quantitative orbital volumetry are recommended to further clarify the relationship between orbital inflammation and mechanical compression.

MRI has a crucial role in orbital soft tissue evaluation and disease progression. Studies have confirmed that enlargement of orbital fat tissue and extraocular muscles compresses the surrounding structures, inducing increased intraorbital pressure and circulatory collapse [11,44]. Studies have confirmed that the thickness of the extraocular muscles in active TED patients is notably higher than in the inactive phase [45,46].

In our hierarchical regression, the positive correlation between TRAb levels and CAS supports the central pathogenic role of TRAb in orbital inflammation, driving fibroblast activation and glycosaminoglycan accumulation [16]. Beyond autoimmunity, our findings emphasize the contribution of systemic metabolic imbalance: elevated triglycerides were independently associated with higher CAS, probably due to oxidative stress, therefore promoting inflammation in GO [47,48]. Serum triglycerides, medial rectus muscle thickness, and retinal microvascular parameters emerged as independent predictors of disease activity, even after adjusting for previous glucocorticosteroid therapy. Increased medial rectus thickness in the model and strong collinearity between other MRI parameters reflect active inflammatory congestion and fibrotic remodeling within the orbit. After inclusion of orbital and microvascular parameters, TRAb lost significance. This likely reflects mediation, as TRAb represents an upstream immunologic trigger whose pathogenic effects manifest through subsequent orbital tissue inflammation, muscle enlargement, and microvascular remodeling [16,49,50]. This model suggests that combined metabolic, retinal microcirculatory and structural MRI changes may serve as a sensitive marker of TED activity. Although earlier reports highlighted total cholesterol and LDL as important contributors to disease activity [51], our multivariate model identified triglycerides as the most robust predictor of CAS, underscoring their potential role in TED-related inflammation.

Several previous studies have demonstrated significant discriminatory ability of OCTA-derived VD in differentiating active from inactive TED [22,52], as well as distinguishing TED from healthy individuals [14,20]. In our study, the ROC analysis further supports the complementary diagnostic contribution of structural and microvascular parameters in TED. While extraocular muscle thickness demonstrated reasonable discriminatory power for identifying active disease, its relatively low specificity suggests considerable overlap between inflammatory enlargement and chronic remodeling, limiting its ability to distinguish active from inactive stages in isolation. In contrast, DCP parafoveal VD provided a slightly higher diagnostic profile. Importantly, the combined model achieved the highest AUC, indicating that integrating microvascular and structural information improves diagnostic precision beyond either parameter alone. Although the discriminative ability was moderate rather than excellent, the observed AUC values indicate potentially useful clinical applicability, particularly in borderline cases where standard clinical scores may be less reliable. Integrating OCTA-derived microvascular metrics with conventional clinical indicators could therefore enhance early recognition of active disease.

This study has several limitations. The cross-sectional nature of the study limits the ability to establish causality and to track the progression of microvascular changes. The sample size was relatively small and the distribution within the two groups was asymmetric due to generally low CAS, which may reduce the statistical power and limit the generalizability of the findings. In addition, patients with poor-quality OCT/OCTA images were excluded from the analysis, which may introduce selection bias and does not fully reflect real-world clinical conditions. The cross-sectional nature of the study also prevents the evaluation of longitudinal changes or disease progression over time. Larger longitudinal studies are needed to determine whether DCP alterations predict disease progression and activity. Finally, all imaging was performed using a single OCTA device and software platform, which may limit the comparability of the results to other imaging systems or technologies.

## 5. Conclusions

Our findings provide evidence of microvascular impairment and its potential association in TED patients. Retinal microcirculation, especially DCP metrics, may serve as a sensitive, noninvasive biomarker reflecting both orbital inflammation and structural remodeling. Integrating OCTA with orbital imaging and metabolic profiling may improve disease activity diagnostics, TED monitoring and potential personalized treatment strategies in TED.

## Figures and Tables

**Table 1:** Demographic and laboratory characteristics of patients with thyroid eye disease (TED).

	Inactive TED (CAS < 3; *n* = 26)	Active TED (CAS ≥ 3; *n* = 12)	*p*-Value *	Hedges’ g/Hodges-Lehmann Estimate	Lower 95% CI	Upper 95% CI
Demographic and clinical parameters						
Female gender	18 (69.23)	9 (75)	0.606			
Age, years	49.31 (9.67)	51.75 (15.02)	0.397 ^†^	−0.21 ^†^	−0.69	0.28
Smoking	15 (57.69)	5 (41.67)	0.193			
Hyperlipidemia ^‡^ (*n* = 29)	7 (35)	6 (66.67)	0.025			
Hypertension	8 (31)	3 (25)	0.787			
TED duration, months	32.5 (24, 90)	16.5 (13.25, 25)	<0.001	18	11	39
GH duration, months	58 (32, 103)	27.5 (16.75, 48.75)	0.002	28	10	54
Radioiodine therapy	3 (11.54)	1 (8.33)	0.67			
Laboratory parameters						
TSH, mIU/L	0.89 (0.004, 1475.0)	0.29 (0.04, 3779.5)	0.201	0.04	−560	0.21
fT3, pmol/L	4.79 (3.94, 9.55)	4.61 (4.42, 6.55)	0.708	0.13	−0.78	0.87
fT4, pmol/L	14.25 (12.04, 20.51)	11.85 (10.16, 14.37)	0.003	3.22	1.02	6.49
anti-TPO, kIU/mL	20.39 (2.07, 363.03)	23.54 (0.61, 1000)	0.968	−0.00	−34.37	5.55
TRAb, IU/L	6.25 (3.7, 9.6)	13.45 (9.45, 21)	<0.001	−6.9	−11.3	−3.2
Total cholesterol ^‡^, mmol/L	4.59 (4.15, 5.68)	5.31 (4.9, 6.77)	0.048	−0.77	−1.47	−0.00
HDL ^‡^, mmol/L	1.3 (1.1, 1.49)	1.47 (1.4, 1.54)	0.007	−0.23	−0.4	−0.07
LDL ^‡^, mmol/L	3.18 (2.68, 4.0)	3.41 (3.02, 4.215)	0.43	−0.39	−1	0.41
Triglycerides ^‡^, mmol/L	1.37 (1.1, 1.8)	2.28 (1.44, 3.5)	0.003	−0.87	−1.75	−0.31

Continuous variables are presented as mean (SD) or median (interquartile range). Categorical variables are presented as a number (percentage). Values reported as 0.00 reflect rounding of values smaller than 0.0001. * Differences between categorical variables tested with Chi-square or Fisher’s exact test, as appropriate. Differences between continuous variables (non-normally distributed) tested with Mann–Whitney U test; effect size reported as the Hodges-Lehmann median difference with 95% CI. ^†^ Differences tested with Student’s *t*-test; effect size reported as Hedges’ g with 95% CI. Effect sizes represent the difference calculated as Inactive TED minus Active TED; negative values indicate higher values in patients with active TED. ^‡^ Lipid profile data were available for 29 par-ticipants. TED, Thyroid Eye Disease; CI, confidence interval; GH, Graves’ hyperthyroidism; TSH, thyroid-stimulating hormone; fT3, free triiodothyronine; fT4, free thyroxine; TRAb, thyrotropin receptor antibody; anti-TPO, anti-thyroid peroxidase antibody; HDL, high-density lipoprotein; LDL, low-density lipoprotein.

**Table 2:** Clinical, angiographic, and MRI characteristics of patients with thyroid eye disease (TED).

	Inactive TED (CAS < 3; *n* = 60 Eyes)	Active TED (CAS ≥ 3; *n* = 16 Eyes)	*p*-Value *	Hedges’ g/Hodges-Lehmann Estimate	Lower 95% CI	Upper 95% CI	*p*-Value GMM
Clinical parameters							
BCVA, Snellen	1 (1, 1)	1 (0.9, 1.0)	0.107 ^†^	0.00 ^†^	−0.00	0.00 ^‡^	0.334
CAS	1 (0, 2)	3.5 (3, 4)	<0.001 ^†^	−3 ^†^	−3	−2	<0.001
Tonometry primary-gaze, mmHg,	14.52 (2.87)	15.25 (2.41)	0.352	−0.26	−0.81	0.29	0.175
Tonometry upward-gaze, mmHg	16.92 (3.81)	21.19 (4.93)	<0.001	−1.04	−1.61	−0.46	0.035
Hertel exophthalmometry, mm	17.2 (3.86)	20 (3.05)	0.011	−0.74	−1.32	−0.16	0.143
OCTA parameters—deep capillary plexus vessel density							
whole image	46.81 (5.53)	50.72 (5.09)	0.013	−0.71	−1.27	−0.15	0.016
fovea	38.03 (7.61)	39.64 (5.24)	0.444	−0.22	−0.79	0.35	0.192
parafovea	53.05 (4.76)	56.4 (3.99)	0.012	−0.72	−1.28	−0.15	0.012
perifovea	47.88 (5.97)	52.31 (5.64)	0.009	−0.74	−1.31	−0.18	0.03
Foveal avascular zone	0.26 (0.09)	0.25 (0.07)	0.789	0.08	−0.48	0.63	0.215
MRI parameters							
Orbital fat-tissue thickness	5.71 (0.97)	6 (1.19)	0.309	−0.29	−0.84	0.27	0.348
Medial rectus muscle thickness	4.5 (4.05, 5.15)	4.85 (4.38, 6.2)	0.103 ^†^	−0.5 ^†^	−1.2	0.1	0.113
Lateral rectus muscle thickness	4.19 (0.76)	4.58 (0.65)	0.083	−0.49	−1.05	0.07	0.212
Superior rectus muscle thickness	4.0 (3.5, 4.75)	4.9 (3.95, 5.85)	0.037 ^†^	−0.8 ^†^	−1.6	0.00	0.042
Inferior rectus muscle thickness	5.79 (1.25)	6.31 (1.41)	0.148	−0.41	−0.96	0.15	0.117
Exophthalmus	18.92 (2.99)	21.01 (4.04)	0.024	−0.64	−1.2	−0.08	0.098

Analyses were performed at the eye level. Continuous variables are presented as mean (SD) or median (interquartile range). Values reported as 0.00 reflect rounding of values smaller than 0.0001. * Differences between continuous variables (normally distributed) tested with Student’s *t*-test; effect size reported as Hedges’ g and 95% confidence intervals (CI). ^†^ Differences tested with Mann–Whitney U test; effect size reported as the Hodges–Lehmann me-dian difference with 95% CI. Effect sizes represent differences calculated as inactive TED minus active TED; negative values in-dicate higher values in eyes with active TED. ^‡^ Due to the bounded nature of Snellen visual acuity, very small effect size estimates should be in-terpreted as indicating no clinically meaningful difference. Generalized estimating models (GMM) were additionally used to calculate *p* values, adjusting for inter-eye correlation within the same patient. TED, Thyroid Eye Disease; BCVA, best corrected visual acuity; CAS, Clinical Activity Score; MRI, magnetic resonance imaging.

**Table 3:** Predictors of parafoveal deep capillary plexus vessel density in hierarchical linear mixed model analysis.

Model	Predictor	B	SE	t	*p*	95% CI Lower	95% CI Upper
M1	Age	−0.11	0.05	−2.12	0.042	−0.21	−0.01
	Sex (male) *	−2.48	1.28	−1.93	0.061	−5.03	0.08
M2	Age	−0.11	0.05	−2.33	0.026	−0.21	−0.02
	Sex (male) *	−2.21	1.23	−1.80	0.081	−4.67	0.24
	CAS	1.17	0.39	3.04	0.003	0.4	1.94
M3	Age	−0.12	0.05	−2.7	0.011	−0.21	−0.03
	CAS	1.42	0.4	3.59	<0.001	0.63	2.21
	Exophthalmos	−0.33	0.16	−2.04	0.046	−0.65	−0.01
	Sex (male) *	−1.97	1.15	−1.71	0.096	−4.27	0.33
Model	Marginal R^2^			*p* ^†^			
M1	0.12			0.016			
M2	0.22			<0.001			
M3	0.26			<0.001			

Hierarchical linear mixed models were used, with eye-level data nested within patients. Regression coefficients (B) are unstandardized estimates. * Negative coefficients indicate lower vessel density in males compared with females. ^†^ Model *p* values indicate overall model significance. SE, standard error; β, standardized regression coefficient; CI, confidence interval; CAS, Clinical Activity Score.

**Table 4:** Hierarchical mixed-effect regression analysis predicting Clinical Activity Score (CAS) in patients with thyroid eye disease (TED).

Model	Predictor	B	SE	t	*p*	95% CI Lower	95% CI Upper
M1	Glucocorticosteroids (yes/no) *	1.06	0.38	2.8	0.008	0.304	1.81
M2	Glucocorticosteroids (yes/no) *	0.54	0.41	1.32	0.202	−0.28	1.36
	TRAb	0.03	0.01	2.77	0.011	0.01	0.05
	Triglycerides	0.43	0.15	2.79	0.011	0.12	0.74
M3	Glucocorticosteroids (yes/no) *	0.33	0.4	0.82	0.42	−0.47	1.12
	TRAb	0.01	0.01	0.59	0.557	−0.02	0.03
	Triglycerides	0.47	0.14	3.30	0.003	0.18	0.76
	Medial rectus muscle thickness	0.38	0.16	2.37	0.022	0.06	0.71
	DCP VD whole image	0.06	0.03	2.30	0.027	0.01	0.12
Model	Marginal R^2^			*p* ^†^			
M1	0.13			0.006			
M2	0.31			<0.001			
M3	0.42			<0.001			

Hierarchical mixed-effects models were fitted with patients as random effects. Regression coefficients (B) represent unstandardized fixed-effect estimates. * Glucocorticosteroid treatment was coded as yes = 1, no = 0. ^†^ Model *p* values indicate overall model significance. SE, standard error; β, standardized regression coefficient; CI, confidence interval; TRAb, thyrotropin receptor antibody; DCP VD, deep capillary plexus vessel density.

## Data Availability

The data supporting the conclusions can be obtained from the corresponding author upon reasonable request and in accordance with ethical and privacy principles.

## Supplementary Materials

The following supporting information can be downloaded at: https://www.mdpi.com/article/10.3390/jcm15010323/s1, Table S1: Correlation coefficients between clinical, laboratory, magnetic resonance imagining (MRI), and optic coherence tomography angiography (OCTA) parameters in patients with thyroid eye disease (TED).

## Abbreviations

The following abbreviations are used in this manuscript:

anti-TPO	anti-thyroid peroxidase antibodies
AUC	area under the curve
BCVA	best-corrected visual acuity
CAS	Clinical Activity Score
CI	confidence interval
DCP	deep capillary plexus
ETDRS	Early Treatment Diabetic Retinopathy Study
EUGOGO	European Society for Graves’ Orbitopathy
FAZ	foveal avascular zone
fT3	free triiodothyronine
fT4	free thyroxine
GH	Graves’ hyperthyroidism
HDL	high-density lipoprotein
ICC	interclass correlation
IOP	intraocular pressure
IQR	interquartile range
LDL	low-density lipoprotein
MRI	magnetic resonance imaging
OCTA	Optical coherence tomography angiography
ROC	receiver operating curve
SCP	superficial capillary plexus
TED	Thyroid eye disease
TRAb	thyrotropin receptor antibodies
TSH	thyroid-stimulating hormone
VD	vessel density
VIF	variance inflation factor
