# Supplementary material for: Retinal Microvascular and Orbital Structural Alterations in Thyroid Eye Disease
Source: J Clin Med. 2026 Jan 1;15(1):323. doi: 10.3390/jcm15010323 (PMC12786960; doi:10.3390/jcm15010323)
Supplement: Supplementary file 1 [file jcm-15-00323-s001.zip › jcm-3997973-supplementary.pdf]

Table S1: Correlation coefficients between clinical, laboratory, magnetic resonance imagining (MRI), and optic coherence tomography angiography (OCTA) parameters in patients with thyroid eye disease (TED).

	DCP VD parafovea	DCP VD perifovea	FTT	MR	LR	SR	IR	Hertel	Cholesterol	Triglycerides	TRAb	TED duration	GH duration	Age	CAS
DCP VD whole image	0.74***	0.98***	-0.07	0.02	-0.19	0.01	-0.14	-0.15	0.09	-0.21	0.12	0.06	0.04	-0.54***	0.09
DCP VD parafovea	1	0.78***	-0.03	0.19	-0.05	0.15	0.04	-0.29	0.13	-0.24	-0.04	0.14	0.04	-0.23	0.27
DCP VD perifovea		1	-0.01	-0.03	-0.20	0.12	-0.11	-0.17	0.13	-0.16	0.1	0.08	0.05	-0.46**	0.08
FTT			1	0.40*	0.15	-0.04	0.36*	0.27	-0.24	0.03	0.15	0.19	0.37*	0.04	0.13
MR				1	0.56***	0.33*	0.60***	0.37*	0.29	0.03	0.30	0.00	0.01	0.01	0.31
LR					1	0.37*	0.32	0.39*	0.42*	0.26	0.16	0.17	0.13	0.29	0.20
SR						1	0.3	0.37*	0.1	-0.05	0.21	0.02	0.03	0.12	0.19
IR							1	0.39*	-0.04	-0.03	0.16	0.26	0.05	-0.01	0.24
Hertel								1	0.00	0.37	0.09	0.09	0.22	-0.08	0.24
Cholesterol									1	0.47*	-0.06	-0.25	-0.29	0.27	0.37*
Triglycerides										1	-0.11	-0.22	-0.25	0.22	0.31
TRAb											1	-0.24	-0.24	0.01	0.05
TED duration												1	0.65***	0.01	-0.21
GH duration													1	-0.05	-0.25
Age														1	-0.03
CAS															

Spearman's correlation coefficients (ρ) are shown. Significant correlations: $p < 0.05$ (*), $p < 0.01$ (**), $p < 0.001$ (***). DCP VD = deep capillary plexus vessel density; FTT = fat tissue thickness; MR, LR, SR, IR = medial, lateral, superior, and inferior rectus muscle thickness; TRAb = thyrotropin receptor antibodies; TED, thyroid eye disease; GH = Graves' hyperthyroidism; CAS = Clinical Activity Score.
